# Image-guided superficial radiation therapy has superior 2-year recurrence probability to Mohs micrographic surgery

**DOI:** 10.1016/j.ctro.2023.100678

**Published:** 2023-09-17

**Authors:** Erin M. McClure, Geoffrey Sedor, Yuxuan Jin, Michael W. Kattan

**Affiliations:** a University of South Florida Morsani College of Medicine, 560 Channelside Dr, Tampa, FL 33602, United States; b Columbia University Irving Medical Center, Vagelos College of Physicians & Surgeons, 630 West 168th Street, New York, NY 10032, United States; c Cleveland Clinic, Dept of Quantitative Health Sciences, Mail Code JJN3, 9500 Euclid Avenue, Cleveland, OH 44195, United States

## Abstract

**Introduction:**

Non-melanoma skin cancers (NMSCs) are the most common cancers in the USA, and their incidence is rising. Mohs micrographic surgery (MMS) is commonly performed to excise NMSCs. MMS replaced superficial radiotherapy (SRT) as a first line treatment, given its superior efficacy. Image-guided superficial radiation therapy (IGSRT) was invented to improve the precision of SRT. This study investigates how the 2-year recurrence probability of IGSRT-treated NMSCs compares to that of MMS-treated lesions.

**Methods:**

This retrospective cohort study compared the 2-year recurrence probability of early stage NMSCs (squamous and basal cell carcinomas (SCCs and BCCs)) treated by IGSRT (2,286 lesions) to data on NMSCs treated by MMS (5,391 lesions) via one sample proportion tests. Medical Subject Headings were used to search PubMed for reports of 2-year recurrence probability rates of NMSCs treated by MMS. Seventeen studies were screened; 14 studies were excluded for lack of 2-year time to event analysis, or irrelevant patient population (non-BCC/SCC study, advanced disease), leaving 3 studies for comparison.

**Results:**

IGSRT-treated NMSCs have a statistically significantly improved 2-year recurrence probability than those treated by MMS, P < 0.001 for pooled data.

**Conclusion:**

The 2-year recurrence probability IGSRT-treated NMSCs is superior to MMS-treated and supports IGSRT as an effective treatment option for individuals with early stage NMSCs.

## Highlights

• 2-year recurrence rate of IGSRT-treated NMSCs is statistically superior to Mohs.
• Statistical superiority supports, at minimum, clinical equivalence.
• This should be considered as a first-line therapy option for NMSCs.
• Well suited for patients who want non-operative therapy or Mohs is contraindicated.

## Introduction

Non-melanoma skin cancers (NMSCs), specifically basal cell carcinomas (BCCs) and squamous cell carcinomas (SCCs), are the most commonly diagnosed cancer in the USA [1]. BCCs and SCCs can be lethal; ∼2000 people die from these cancers each year, and they are a significant cause of morbidity [2], [3]. In 2012, there were 5.4 million cases of NMSCs, and the incidence is increasing ∼ 2% annually in the USA [4], [5].

Current National Comprehensive Cancer Network (NCCN) guidelines for the treatment of localized but high-risk BCCs and SCCs include surgical excision or Mohs micrographic surgery (MMS) [6], [7]. If patients are poor surgical candidates, radiation therapy is recommended [6], [7].

Superficial radiation therapy (SRT) is a type of radiation utilizing low energy kilovoltage photons (50-150kVp) to confine treatment to the skin [8]. Typical dosing schedules for NMSCs range from 5 Gy × 7 fractions (35 Gy total) to 2 Gy × 30 fractions (60 Gy) [9]. Higher total doses are administered to younger patients or larger tumors. In contrast, lower total doses are used in patients with major comorbidities or of advanced age [9]. SRT was widely used in the 1970′s to treat early stage NMSCs and was relegated to second line because of the improved oncologic outcomes of MMS [10].

Image-guided SRT (IGSRT) utilizes high frequency ultrasound (22 MHz) to visualize skin cancers more precisely [11]. The 2-year recurrence probabilities of IGSRT-treated NMSCs were compared to previously documented 2-year recurrence probabilities of SRT. IGSRT had a statistically superior pooled (BCC + SCC + SCCIS) 2-year recurrence probability of 0.7% (p < 0.001) to SRT [12]. IGSRT also outperformed SRT when NMSC 2-year recurrence probability was stratified by histology [12]. Given the improved 2-year recurrence probabilities of IGSRT-treated NMSCs to SRT-treated, this study aims to investigate how IGSRT compares to MMS since MMS has historically replaced SRT as a first-line therapy. This is the first study to perform this head-to-head comparison.

## Methods

Ethics committee/IRB of WCG IRB waived ethical approval for this work. WCG IRBs IRB Affairs Department reviewed the study under the Common Rule and applicable guidance. The response stated this study is exempt under 45 CFR | 46.104(d)(4), because the research involves the use of identifiable private information; and information is recorded by the investigator in such a manner that the identity of the human subjects cannot readily by ascertained directly or through identifiers linked to the subjects, the investigator does not contact the subjects, and the investigator will not re-identify subjects.

### Pooled analysis method

In addition to individual comparisons by histology of reported groups, an overall, pooled outcome comparison was desired. To conduct this comparison of the IGSRT cohort to those reported in the literature on MMS, results from three reference groups which reported enough granularity of outcome to compute 2-year recurrence probabilities were pooled. Then, the total number of events and total number of patients for the combined cohort were summed for comparison to the IGSRT data. The recurrence rate for patients in the reference groups across with SCC (which was included in all 3 published groups cohorts) was calculated using the weighted average of the recurrent rate from each study whose weight was calculated based on its sample size relative to the total sample size combining the three studies. The IGSRT recurrence probabilities and these pooled estimates were compared using a test of proportions.

### Pubmed search strategy

The following search strategy utilizing Medical Subject Heading (MeSH) terms was used in Pubmed to find relevant literature:

(((“Follow up studies”[Mesh] OR “Neoplasm recurrence, local”[Mesh] or “Survival rate”[Mesh] or “treatment outcome”[Mesh] or recurrence rate) and “2 year” OR “two year”) AND (“Mohs surgery”[Mesh] or Mohs or mohs micrographic surgery)) AND (“Skin Neoplasms”[Mesh] or “Carcinoma, Basal Cell/pathology”[Mesh] or “Carcinoma, Basal Cell/surgery”[Mesh] or “Carcinoma, Basosquamous”[Mesh] or “non-melanoma skin cancer” or “squamous cell carcinoma” or “basal cell carcinoma”).

**Fig. 1:**
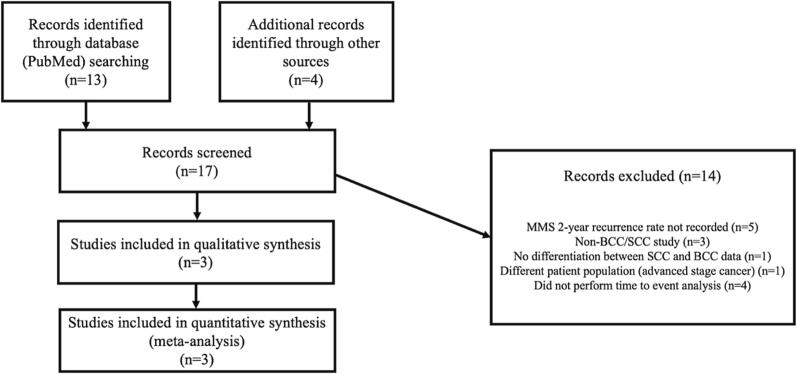
Flow diagram for article selection.

Seventeen studies were identified in total, and after thorough screening for relevance, 3 studies were included in this *meta*-analysis, see Fig. 1 for exclusions. A list of all studies screened can be found in Supplemental Table 1. As a result of this search strategy, MMS comparisons were sourced from the following papers: Tomás-Velázquez et al 2021, Xiong et al 2020, and Van Lee et al 2019 [13], [14], [15].

### Image-guided superficial radiation therapy data sourcing

IGSRT-treated NMSC data is from a recently published database, from which the 2-year recurrence probability of IGSRT-treated NMSCs has been determined [11], [12]. IGSRT 2-year probabilities were compared with those of MMS in the literature with a test of proportions.

## Results

**Fig. 2:**
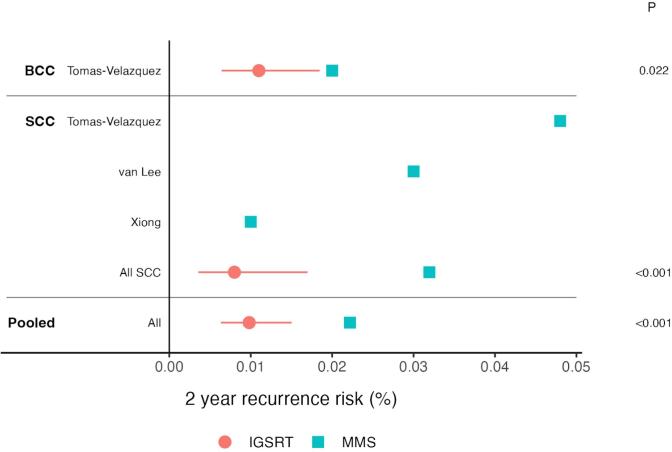
IGSRT has a statistically lower 2-year recurrence probability than MMS in NMSCs (BCC and SCC). Red circles represent IGSRT data and blue squares represent MMS data. Red lines represent 95% confidence intervals for IGSRT recurrence rates as calculated from the test of proportions.

Three studies reported on SCC 2-year recurrence probabilities after treatment by MMS and one study reported on BCCs; see Table 1. Two-year recurrence probabilities of all reported SCCs (989 lesions total) and BCCs (4,402 lesions) treated by MMS were pooled and also stratified by histology. Pooled IGSRT-treated NMSCs had a statistically significantly improved 2-year recurrence probability to pooled MMS-treated lesions (p < 0.001), and to MMS-treated lesions separated by histologic type (SCCs p < 0.001, and BCCs p = 0.022), see Fig. 2.

**Table 1:** Summary of studies reporting the 2-year recurrence probabilities of SCCs and/or BCCs treated by MMS or IGSRT.

Authors, year	PMID	Disease	Study design	Treatment modality	Cohort (n/age/sex)	2-year recurrence probability
Alejandra Tomas-Velazquez, 2021	34,694,418	SCC, BCC	Prospective cohort conducted in 22 Spanish centers and a multivariate analysis	MMS	n = 371 SCC n = 4,402 BCC	0.048 for SCC 0.020 for BCC
C.B. van Lee, 2018	30,199,574	SCC	Retrospective multi-institution (2) cohort study	MMS	n = 380 262 men, 118 women median age 76 (IQR 69–81)	0.030
David D. Xiong, 2020	31,887,322	SCC	Retrospective single institution chart review	MMS	n = 238	0.010
Erin McClure, 2022		SCC, BCC	Retrospective cohort study	IGSRT	BCC (N = 1382) SCC (N = 904)	BCC 0.011 SCC 0.008

## Discussion

### Major findings

There is a statistically significant improvement in 2-year recurrence probabilities of NMSCs when treated primarily with IGSRT compared to MMS. This was observed both in pooled NMSC data and when stratified by histological type (BCC, SCC). This suggests that IGSRT should become a first-line recommendation for patients with early stage NMSCs like BCCs, and SCCs, particularly for individuals who are poor candidates for or refuse surgical resection.

### Advantages to IGSRT

IGSRT is well tolerated. In a recent study, Radiation Therapy Oncology Group (RTOG) toxicity grade data was reported on 75% (2177/2917) of IGSRT-treated lesions [11]. Approximately 80% of these adverse events were grade 1 cutaneous toxicities, and ∼ 20% were grade 2, and they typically self-resolved within 2 weeks [11].

Each procedure visit is relatively short (visits are 10–15 min/lesion – treatment itself is < 1 min) whereas a typical MMS takes 2–4 h [16]. IGSRT eliminates the concern for wound infection, dehiscence, bleeding, hematoma formation. For example, the risk of developing keloids following a surgical procedure is eliminated. This therapy is an option for patients with contraindications to surgery like certain cardiac conditions or coagulopathies. Lastly, treatment with IGSRT can include up to 4 lesions at one time (average is 1.7 lesions), and treatment is not constrained by anatomy.

IGSRT results in favorable cosmetic outcomes, without scarring from this non-invasive procedure [17]. Therefore, reconstructive/scar revision procedures are avoided. This is particularly notable as NMSC as the greatest incidence on sun exposed areas such as the head and neck, which are cosmetically sensitive. Furthermore, the NCCN guidelines for BCC and SCC both state in the principles of treatment sections, “the primary goal of treatment is the complete removal of the tumor and the maximal preservation of function and cosmesis.” [6], [7]. The NCCN guidelines for BCC specifically reference the use of “Radiation Therapy (RT) for non-surgical candidates” diagnosed with either low-risk or high-risk BCC [6]. Similarly, the NCCN guidelines for SCC note that while surgery may be effective and efficient, “considerations of function, cosmesis, and patient preference may lead to choosing RT as primary treatment in order to achieve optimal overall results.” [7]. The NCCN-SCC guidelines go on to state that “all treatment decisions should be customized to account for the particular factors present in the individual case and for the patient’s preference.” Therefore, according to the NCCN guidelines, radiation therapy such as IGSRT is an effective treatment option for patients who refuse surgery and maximal preservation of function and cosmesis is considered, noting that local control rates for radiation therapy are near 100%.

### Advantages to MMS

While there are clearly advantages to IGSRT, MMS has its own benefits. MMS ensures the cancer procedure is completed in a single visit, (in most cases). Notably, this one-day therapy is particularly convenient for patients who reside far away from treatment facilities. MMS is widely performed, and relatively easily accessible by patients. Additionally, patients learn the same day of treatment that their cancer is cured, which can relieve psychological stress. See Table 2 for a shared decision-making tool.

**Table 2:** An option gride designed to help physicians and patients come to a shared decision on how to treat non-melanoma skin cancer. Frequently asked questions sourced from cancer.org[19].

Frequently asked questions	Image-guided superficial radiation therapy	Mohs micrographic surgery
What will treatment be like?	Treatment is prescribed and performed in an outpatient dermatology or radiation oncology clinic. Treatment delivery is performed by a radiation therapist. The therapy is administered over 4–7 weeks, with 3–5 sessions/week. Each session lasts ∼ 15 min. Up to 3 skin cancers can be treated simultaneously. First, gel is applied to the treatment area and an ultrasound wand is placed over the gel to better visualize the cancer. The width, depth and breadth of the tumor is calculated, then the arm of the radiation device is placed over the treatment site and X-rays are precisely and painlessly delivered to the tumor (no need for anesthesia) .	Patients are typically awake for this procedure. A trained Mohs surgeon will clean and numb the operation site. The tumor will be cut out and examined under the microscope. The patient is bandaged and waits for completion of the microscopic examination. If the microscopic exam identifies tumor at the edges of the removed tissue, the surgeon will cut away another layer of skin from the patient and re-examine. This repeats until the tissue edges do not show cancer. The patient’s wound is either stitched closed or they undergo reconstructive surgery for more extensive operations [19].
What are the risks/side effects from treatment?	Side effects usually self-improve quickly, within 2–6 weeks after treatment. The most common symptoms are mild to moderate skin irritation and redness. These are primarily controlled with over-the-counter ointments/creams. This surgery-free treatment does not require cutting and therefore there is no bleeding, no surgical pain with treatment delivery, and no need for reconstructive or scar revision procedures.	There is a small risk of bleeding, infection, nerve damage, tumor recurrence, tissue necrosis, and re-opening of the wound [17].
Will there be a scar after treatment?	There will not be any surgical scarring after treatment because there is no surgical wound. However, patients may experience a change in color of the skin within the radiation field.	There will be a scar after treatment since this involves a surgical procedure [17]. Size of the scar varies on a case-by-case basis. Reconstructive surgery and size of the tumor can impact size and location of the scar(s).
What should I do to be ready for treatment?	A consultation with a dermatologist occurs before treatment. Patients can continue their normal daily activities before and after treatment. Patients will not need to stop blood thinners or take antibiotics after treatment.	A pre-operative evaluation of the patient’s current state of health, past medical history, and medications is required. Some high-risk patients are prescribed prophylactic antibiotics [17]

### Study limitations

Beyond histology, cohort matching was unable to be performed due to missing data (tumor size, stage) in comparison papers. Previously detailed reports of the IGSRT cohort including tumor size, location, and stage distributions can be found in Supplemental Tables 2 and 3, which will hopefully be of use to future comparisons [12]. The lack of comprehensive cohort matching increases the possibility that confounding factors are impacting the statistical analysis. For example, it is possible that the IGSRT cohort had a higher proportion of lower risk lesions than those in the MMS comparison cohorts, which could improve the 2-year recurrence probability of IGSRT-treated lesions. Another limitation is the retrospective nature of this study, as correlations can be made, but causations cannot. Lastly, 5-year recurrence rates are a common end point in evaluating treatment efficacy of BCCs and SCCs; however, more time must pass before a 5-year analysis can be done since IGSRT is a relatively new treatment.

### Future directions

To address a study limitation, we aim to repeat this analysis once sufficient data of recurrence rates of NMSCs at the 5-year mark following IGSRT has been collected. Another goal is to repeat this analysis with more robust cohort matching if a study is published with detailed data on the stage and size of NMSCs treated by MMS.

The field of individualized medicine continues to rapidly develop. Studies have determined personalized radiation regimens based on a genomic-adjusted radiation dose (GARD) [20], [21]. A higher GARD is associated with an improved outcomes. GARD independently predicted clinical outcomes (time to first recurrence and overall survival) of patients of various cancer types (including NMSCs) treated by radiation. This may be the key to the future of individualized radiation treatment plans, including IGSRT, and how to select patients best suited for MMS or IGSRT.

## Conclusion

In summary, in this analysis IGSRT outperforms MMS in the treatment of NMSCs as determined by a statistically significantly superior 2-year recurrence probability. There is a subset of patients (those who refuse MMS, have contraindications to surgery, or prefer the toxicity profile of IGSRT) where this is a unique and advantageous treatment modality. It is important to provide patients as many effective treatment options as possible. Patients should be empowered to participate in their treatment course during the full informed consenting process, which affirms the patient’s role in the decision-making process. The data in this study is compelling and supports the continued practice, and expansion of IGSRT.

## Funding

None.

## CRediT authorship contribution statement

**Erin McClure:** Conceptualization, Methodology, Data curation, Writing – original draft. **Geoffrey Sedor:** Visualization, Writing – review & editing. **Yuxuan Jin:** Formal analysis, Writing – review & editing. **Michael W. Kattan:** Supervision, Writing – review & editing, Methodology.

## Declaration of Competing Interest

Dr. Kattan is a paid consultant for Skin Cure Oncology. Remaining authors have no conflicts of interest to disclose.
