# Corrigendum to “Image-guided superficial radiation therapy has superior 2-year recurrence probability to Mohs micrographic surgery” [Clin. Transl. Radiat. Oncol. 43 (2023) 100678]

**DOI:** 10.1016/j.ctro.2024.100876

**Published:** 2024-10-23

**Authors:** Erin M. McClure, Geoffrey Sedor, Yuxuan Jin, Michael W. Kattan

**Affiliations:** a University of South Florida Morsani College of Medicine, 560 Channelside Dr, Tampa, FL 33602, United States; b Columbia University Irving Medical Center, Vagelos College of Physicians & Surgeons, 630 West 168th Street, New York, NY 10032, United States; c Cleveland Clinic, Dept of Quantitative Health Sciences, Mail Code JJN3, 9500 Euclid Avenue, Cleveland, OH 44195, United States; d University Hospitals Geauga Medical Center, 13207 Ravenna Rd, Chardon, OH 44024, United States

The authors regret to share that Dr. Michael Kattan, the corresponding author and senior statistician for this work, sadly passed away. Dr. Erin McClure is now the corresponding author.

