# Supplementary material for: Image-guided superficial radiation therapy has superior 2-year recurrence probability to Mohs micrographic surgery
Source: Clin Transl Radiat Oncol. 2023 Sep 17;43:100678. doi: 10.1016/j.ctro.2023.100678 (PMC10539860; doi:10.1016/j.ctro.2023.100678)
Supplement: Supplementary data 1 [file mmc1.docx]

**Supplemental Table 1.** Literature evaluated for suitability for statistical analysis.

| **Pubmed search articles** |
| --- |
| 1. Macfarlane, Louise, et al. "Seven years' experience of Mohs micrographic surgery in a UK centre, and development of a UK minimum dataset and audit standards." Clinical and Experimental Dermatology 38.3 (2013): 262-269. |
| 1. Xiong, David D., et al. "Outcomes in intermediate-risk squamous cell carcinomas treated with Mohs micrographic surgery compared with wide local excision." Journal of the American Academy of Dermatology 82.5 (2020): 1195-1204. |
| 1. Van Lee, C. B., et al. "Recurrence rates of cutaneous squamous cell carcinoma of the head and neck after Mohs micrographic surgery vs. standard excision: a retrospective cohort study." British Journal of Dermatology 181.2 (2019): 338-343. |
| 1. Tomás-Velázquez, Alejandra, et al. "Risk factors and rate of recurrence after Mohs surgery in basal cell and squamous cell carcinomas: a nationwide prospective cohort (REGESMOHS, Spanish registry of Mohs surgery)." Acta Dermato-Venereologica 101.11 (2021). |
| **Additional records** |
| 1. Soleymani, Teo, et al. "Clinical outcomes of high-risk cutaneous squamous cell carcinomas treated with Mohs surgery alone: An analysis of local recurrence, regional nodal metastases, progression-free survival, and disease-specific death." Journal of the American Academy of Dermatology 88.1 (2023): 109-117. |
| 1. Allen, Kattie J., et al. "Basosquamous carcinoma and metatypical basal cell carcinoma: a review of treatment with M ohs micrographic surgery." International journal of dermatology 53.11 (2014): 1395-1403. |
| 1. Calvão, Joana, et al. "Clinicopathological factors influencing the number of stages of Mohs surgery for basal cell carcinoma." Anais Brasileiros de Dermatologia 97 (2022): 291-297. |
| 1. Patel, Arsh, et al. "Outcomes of adjunctive therapies post hedgehog inhibitors in the management of locally advanced basal cell carcinoma: a systematic review and pooled analysis." Dermatologic Therapy 34.6 (2021): e15172. |
| 1. Zhang, Junbo, et al. "Clinical efficacy of Mohs surgery combined with topical photodynamic therapy for facial basal cell carcinoma." Journal of Cancer Research and Therapeutics 16.5 (2020): 1051-1055. |
| 1. Manyam, Bindu V., et al. "A multi‐institutional comparison of outcomes of immunosuppressed and immunocompetent patients treated with surgery and radiation therapy for cutaneous squamous cell carcinoma of the head and neck." Cancer 123.11 (2017): 2054-2060. |
| 1. Chow, William, et al. "Growth attenuation of cutaneous angiosarcoma with propranolol-mediated β-blockade." JAMA dermatology 151.11 (2015): 1226-1229. |
| 1. Ibrahimi, Omar A., et al. "Repair of Scalp Defects Using an H‐Plasty Type of Bilateral Advancement Flap." Dermatologic surgery 36.12 (2010): 1993-1997. |
| 1. Silapunt, Sirunya, S. Ray Peterson, and Leonard H. Goldberg. "Squamous cell carcinoma of the auricle and Mohs micrographic surgery." Dermatologic surgery 31.11 (2005): 1423-1427. |
| 1. Snow, Stephen, et al. "Microcystic adnexal carcinoma: report of 13 cases and review of the literature." Dermatologic surgery 27.4 (2001): 401-408. |
| 1. Rudolph, Ross, and Stephen H. Miller. "Reconstruction after Mohs cancer excision." Clinics in plastic surgery 20.1 (1993): 157-165. |
| 1. Niparko, John K., et al. "Local control of auricular, periauricular, and external canal cutaneous malignancies with Mohs surgery." The Laryngoscope 100.10 (1990): 1047-1051. |
| 1. Rowe, Dan E., RAYMOND J. CARROLL, and CALVIN L. JR DAY. "Long‐term recurrence rates in previously untreated (primary) basal cell carcinoma: implications for patient follow‐up." The Journal of dermatologic surgery and oncology 15.3 (1989): 315-328. |

**Supplemental Table 2.** Clinical characteristics stratified by histology.

| **Characteristic** | **BCC, N = 1,382** | **SCCis, N = 594** | | **SCC, N = 904** |  |
| --- | --- | --- | --- | --- | --- |
| Stage | | | | |  |
| 0 | 0 | 594 | | 0 |  |
| 1 | 1,150 | 0 | | 746 |  |
| 2 | 232 | 0 | | 158 |  |
| Event | | | | |  |
| Death (other cause) | 30 | 11 | | 29 |  |
| Recurrence | 14 | 1 | | 7 |  |
| Follow Up (months)* | 23.3 (8.3, 35.8) | 32.9 (15.1, 43.1) | 28.3 (11.4, 38.4) | | |

N; *Median (IQR)

**Supplemental Table 3.** Tumor sites included in our cohort of patients treated with IGSRT, stratified by histology. Staging and events by histology.

| **Site** | **BCC, N = 1,382** | **SCCis, N = 594** | **SCC, N = 904** | **Total, N = 2,880*** |
| --- | --- | --- | --- | --- |
| High-risk sites^ | 611 | N/A | 168 | 779 |
| Head and Neck (H&N) | 1,003 | 371 | 537 | 1911 |
| *H&N sublocation* | | | | |
| Ear | 121 | 54 | 95 | 270 |
| Scalp | 52 | 58 | 83 | 193 |
| Forehead | 104 | 74 | 73 | 251 |
| Temple | 37 | 12 | 19 | 68 |
| Forehead/Temple | 0 | 1 | 0 | 1 |
| Eyebrow | 5 | 4 | 2 | 11 |
| Eyelid | 24 | 1 | 2 | 27 |
| Nose | 350 | 46 | 78 | 474 |
| Cheek | 191 | 92 | 120 | 403 |
| Cutaneous Lip | 48 | 7 | 13 | 68 |
| Mucosal Lip | 3 | 4 | 13 | 20 |
| Chin | 6 | 0 | 0 | 6 |
| Jawline | 2 | 0 | 3 | 5 |
| Chin/Jawline | 1 | 0 | 0 | 1 |
| Neck | 59 | 18 | 35 | 112 |
| Other | 0 | 0 | 1 | 1 |
| Extremities | 164 | 181 | 304 | 649 |
| *Extremities sublocation* | | | | |
| Hand | 11 | 53 | 78 | 142 |
| Other | 153 | 128 | 226 | 507 |
| Shoulder | 60 | 10 | 15 | 85 |
| Trunk | 152 | 31 | 46 | 229 |
| *Trunk sublocation* | | | | |
| Chest | 45 | 10 | 26 | 81 |
| Back | 96 | 21 | 20 | 137 |
| Other | 11 | 0 | 0 | 11 |
| Penis | 0 | 0 | 1 | 1 |

*5 lesion sites not recorded.
^High-risk anatomical sites for BCC include nose, paranasal folds, periocular, scalp, temples, and lips. High risk sites for SCC include lips, ears, and non-sun exposed skin.[22]
